# Subacromial triamcinolone acetonide, hyaluronic acid and saline injections for shoulder pain an RCT investigating the effectiveness in the first days

**DOI:** 10.1186/1471-2474-15-352

**Published:** 2014-10-23

**Authors:** Ludo IF Penning, Rob A de Bie, Geert HIM Walenkamp

**Affiliations:** Department of Orthopaedic Surgery, Maastricht University Medical Centre, P. Debeyeplein 25, PO Box 5800, 6202 AZ Maastricht, The Netherlands; Department of Orthopaedic Surgery, Sint Maartenskliniek, Hengstdal 3, PO Box 9011, 6500 GM Nijmegen, The Netherlands; Research Institute Caphri, Maastricht University, PO Box 6166200MD, Maastricht, The Netherlands; Department of Epidemiology Maastricht University, P.Debeyeplein 1, PO Box 6166200MD, Maastricht, The Netherlands

**Keywords:** Subacromial impingement, Pain diary, Subacromial injection, Corticosteroid, Hyaluronic acid, Placebo

## Abstract

**Background:**

Subacromial impingement is a common cause of shoulder complaints in general practice. When the initial treatment with acetaminophen and low dose Non Steroidal Anti Inflammatory Drugs fails, triamcinolone acetonide injections are commonly used. Triamcinolone acetonide injections are effective at four to six weeks. Little is known about the pain relief effect of triamcinolone acetonide injections in the first days after injection and the effect of repeated injection. In this study we investigate the effect of triamcinolone acetonide injections compared to hyaluronic acid and NaCl injections using a pain diary.

**Methods:**

159 Patients recruited for an RCT comparing the effect of subacromial injections of triamcinolone acetonide, hyaluronic acid and sodium chloride (NaCl) were used in this study. They were blinded for their treatment and could receive up to three injections. Primary outcome consisted of the patient perceived pain on a VAS score recorded on a daily basis during 21 days following injection. Secondary outcome consisted of the amount of taken escape medication following injection and adverse effects.

**Results:**

All patients received the first injection. 150 patients also received the second and third injections. 97% Of the paper and pencil pain diaries were returned for data analysis.

The triamcinolone acetonide group showed the largest decrease in pain on the VAS scores after injection compared to the hyaluronic acid and NaCl group in the first week after injection. The reduction in pain was best achieved after the first injection, the second triamcinolone acetonide injection showed a further reduction in pain. The third triamcinolone acetonide injection only showed a slight improvement in pain reduction.

**Conclusions:**

In this study we could show a booster effect in pain reduction after repeated triamcinolone acetonide injection. The triamcinolone acetonide group showed a faster reduction in pain after injection compared to the hyaluronic acid and NaCl group. The effect was best seen after the first and second triamcinolone acetonide injection, it is therefore questionable whether it is necessary to repeat triamcinolone acetonide injections more than two times.

**Trial registration:**

ISRCTN51511455. Registered 20 December 2005

**Electronic supplementary material:**

The online version of this article (doi:10.1186/1471-2474-15-352) contains supplementary material, which is available to authorized users.

## Background

Shoulder complaints are frequently encountered in General Practice [1, 2], of which subacromial impingement is a common cause [3]. Early symptoms of subacromial impingement are treated with acetaminophen (paracetamol) or low-doses of non-steroidal anti-inflammatory drugs. When this therapy fails, impingement can be treated with subacromial injections. These injections merely consist of a combination of a corticosteroid and a local anesthetic [4, 5]. Although it is known that corticosteroids are effective at short term, four to six weeks, only a few studies have investigated the effect of corticosteroids in the first days after injection. In the study of Lewis this was investigated for the treatment in lateral epicondylitis of the elbow in the review of Coombs this consisted of the study of the effectiveness of injections in a number of different tendinopathy pathologies like lateral epicondylalgia, medial epicondylalgia, rotator-cuff tendinopathy, Achilles tendinopathy and patellar tendinopathy [6, 7]. These drug treatments are commonly combined with exercises and physiotherapy, the combination of subacromial injections and physiotherapy are stated to give a better result [8].

A few studies have shown improvement in pain and function following the subacromial injection of hyaluronic acid alone [9–12].

Hyaluronic acid is thought to serve as a lubricant [13] and is reported to have an anti-inflammatory effect [14].

Several studies have investigated the side effect of corticosteroid injections, in these studies only few side effects are described provided injections are not repeated too often at the same site [15]. Some animal studies reported negative side effects concerning weakening of the rat rotator cuff in the first weeks after administration [16–18]. The corticosteroid injection reduces the inflammation and pain associated with the subacromial impingement [19].

Little is known about the effect on pain reduction after repeated corticosteroid injection [20, 21].

This study was part of a randomized clinical trial RCT, with a three arm design in which hyaluronic acid + lidocaine 1% (A) were compared to triamcinolone acetonide + lidocaine 1% (B) and placebo sodium chloride (NaCl) + lidocaine 1% (C). In this trial we could prove a significant improvement in pain for triamcinolone acetonide after three, six and twelve weeks. Compared to placebo, injections containing hyaluronic acid did not show a significant improvement in pain [22].

In this study we investigated the effect of subacromial injections in the first days after administration. We also investigated the effect of repetition of subacromial injections on the perceived amount of pain in order to study an accumulative effect of repeated injection.

## Methods

### Setting and participants

This study was approved by the Medical Ethics Review Committee (MERC) of the Maastricht University Medical Center (MUMC) and was performed at the outpatient clinic of our orthopedic surgery department. Written informed consent was obtained from participants. A total of 159 patients were included in the study, including 75 men and 84 women with a mean age of 53 years (range 20 to 87). A large majority of patients could be included after direct referral by general practitioners. These patients did not receive prior injection treatment by their general practitioners for their current episode of shoulder pain.

Patients were randomized into three treatment groups and were blinded for their respective treatment. Eligible patients were over 18 years of age and had pain in the shoulder, either at rest or on movement. The diagnosis of impingement was made clinically without the routine use of ultrasound or MRI. All presented with a painful arc, with or without abnormal scapulohumeral movement.

Exclusion criteria included: pain for less than six weeks; injection with corticosteroids in the preceding three months; flexion of <100° in the frontal plane; external rotation limited by >50% compared with the opposite side; allergy to lidocaine 1%, steroids or hyaluronic acid; pregnancy or suspected pregnancy; dementia; prior infection of the shoulder joints; tumour; osteoporosis; rheumatoid arthritis according to the American College of Rheumatology (ACR) criteria [23]; referred pain, such as from the neck; an associated neurological disorder; polymyalgia; ankylosing spondylitis as diagnosed using the modified New York (NY) criteria [24]; whiplash injury; previous fractures or surgery on the shoulder, upper limb, neck or thorax; and behavioural, cognitive or psychiatric disorders. Patients unable to complete Dutch questionnaires independently or reluctant to adhere to the allocated treatment or to complete follow-up were also excluded [22].

### Intervention

Treatment consisted of subacromial injection with either: a mixture of 8 ml lidocaine 1% and 2 ml hyaluronic acid (Ostenil) (TRB CHEMEDICA, Haar/München, Germany) (group A); a mixture of 8 ml lidocaine 1% with 2 ml triamcinolone acetonide 10 mg/ml (group B); or a mixture of 8 ml lidocaine 1% with 2 ml NaCl 0.9% (placebo; group C). Injections were repeated, if necessary, after three and six weeks. In case of complete resolution of pain, no further injections were given [22].

### Randomization

Patients were randomized blindly into three treatment groups. An independent statistician (FK) generated a random numbers list which, by permutation of random blocks, block size 9, was balanced for treatments within strata. Strata were based on age (≤40 years *versus* >40 years). After selection and baseline assessment, consecutive numbered opaque envelopes of the appropriate stratum were opened by one of several independent trial nurses.

A total of 51 patients were randomized into group A, 53 into group B and 55 into group C [22].

### Blinding

All injections were administered by the same physician (LIFP). Both physician and patients were blinded to the contents of the syringe. In order to achieve an effective blinded injection a 19 gauge 1.5 inch needle and a 10 ml syringe were used to prevent the physician identifying the difference in viscosity of the administered solutions. The syringes were filled by an independent trial nurse and masked with black adhesive tape. The nurse was thus responsible for the blinding procedure. Inclusion, follow-up assessments and data analysis were blinded for allocated treatment [22].

### Administration of injection

Injections were administered via a dorsolateral approach through the interval just beneath the dorsal acromial edge, with the patient sitting up [25]. The injection site was marked and disinfected with iodine, or with chlorhexidine solution in case of known allergy.

### Outcome assessment

The primary outcome measure was pain as measured on a horizontal 10 cm visual analogue scale (VAS) (0, no pain to 10, severe pain) [26]. Measurements were taken at consultation before injection. The first 21 days after each injection patients were asked to record a VAS score each day in their pain diary.

We investigated the effect of injections of A, B and C on a daily basis using a pain diary during the 21th days after injection. In this study we focus on the effects of repeated triamcinolone acetonide injections versus placebo and hyaluronic acid, illustrating the potential benefit of a series of injections.

The pain diary was used to record the perceived amount of pain. With the pain diary we are able to show the immediate daily response the first days after injection. Pain diaries have been shown to be valid and reliable in the measurement of pain in patients with chronic pain and cancer. It is reported that patients tend to overestimate their remembered pain and psychosocial and pain related factors tend to bias the remembered pain. The daily self-recording of pain by a pain diary showed to reduce the amount of bias [27].

Secondary outcome measures consisted of the type and amount of escape medication taken and the occurrence of adverse effects.

During the first visit patients were thoroughly instructed how to fill out the paper and pencil pain diary. Patients were told to record the maximum experienced pain for each day.

In the pain dairies the days were separated. For each day a VAS score could be recorded on a 10 cm line.

Although patients were instructed that only escape medication consisting of acetaminophen (paracetamol) was allowed, they were asked to record the type and amount of escape medication taken for each day.

A final question for each day consisted of the occurrence of adverse effects. The perceived adverse events were recorded on a text free basis.

Patients were instructed to complete the diary on a daily basis, they were suggested to fill out the diary at the same moment each day to improve compliance.

### Statistical analysis

For this study we obtained data of patients enrolled in the RCT. The number of participants was based on the power calculation used for the RCT in which we had calculated a total number of 159 participants, 53, per group, allowing for a dropout rate of 10%. The power calculation of the RCT was based on the decrease in pain on a VAS score [22].

First, data were tested for normality. Second, a missing value analysis was performed. In case of loss to follow-up or withdrawal resulting in missing data, these values were handled by the method of linear trend at point imputation. Analysis of variance was used to establish effects of both interventions. The influence of prognostic variables and baseline differences for outcome measures was assessed in a multivariate linear regression model with imputation of most important baseline variables. Influential variables were used to correct the outcome of the analysis of variance. Statistical significance was set at a p-value <0.05 [22].

## Results

**Figure 1:**
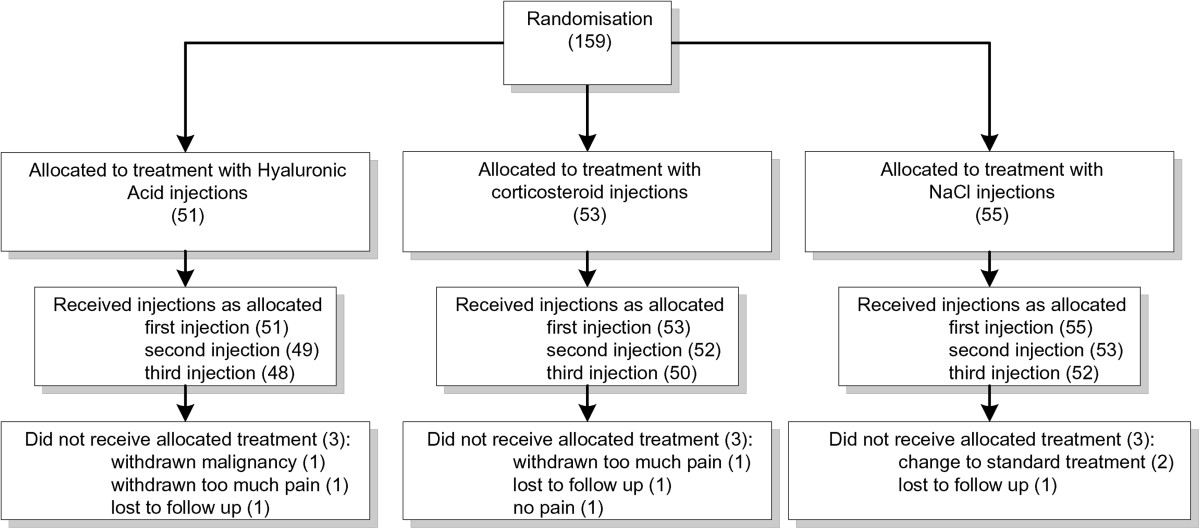
**Allocated treatment.**

All of the 159 included patients received the first allocated injection. The second and third allocated injections were received by 150 patients. Nine patients (6%) did not receive the second and third injection, 3 in each of the groups. In the hyaluronic acid group reasons for not receiving the allocated injections were: withdrawn because of malignancy (n = 1), lost to follow up (n = 1) and not wishing to continue (n = 1). In the triamcinolone acetonide group reasons for not receiving the allocated treatment were: withdrawn because of too much pain (n = 1), lost to follow up (n = 1) and complete relieve of pain after 2 injections (n = 1). In the NaCl injection group reasons for not receiving the allocated treatment consisted of too much pain (n = 2) and lost to follow up (n = 1). [22] Figure 1.

After each injection the result of the Neer test was recorded [28]. In the hyaluronic acid group we report a positive Neer test in 61%, in the triamcinolone group in 70,5% and in the NaCl group in 73%.

The analysis at baseline did not show any significant differences among the groups. At baseline the VAS score of the triamcinolone acetonide group was 5.8 (95% CI 5.1-6.5), of the hyaluronic acid group 6.1(95% CI 5.4-6.8) and of the NaCl group was 5.9 (95% CI 5.1-6.5).

**Figure 2:**
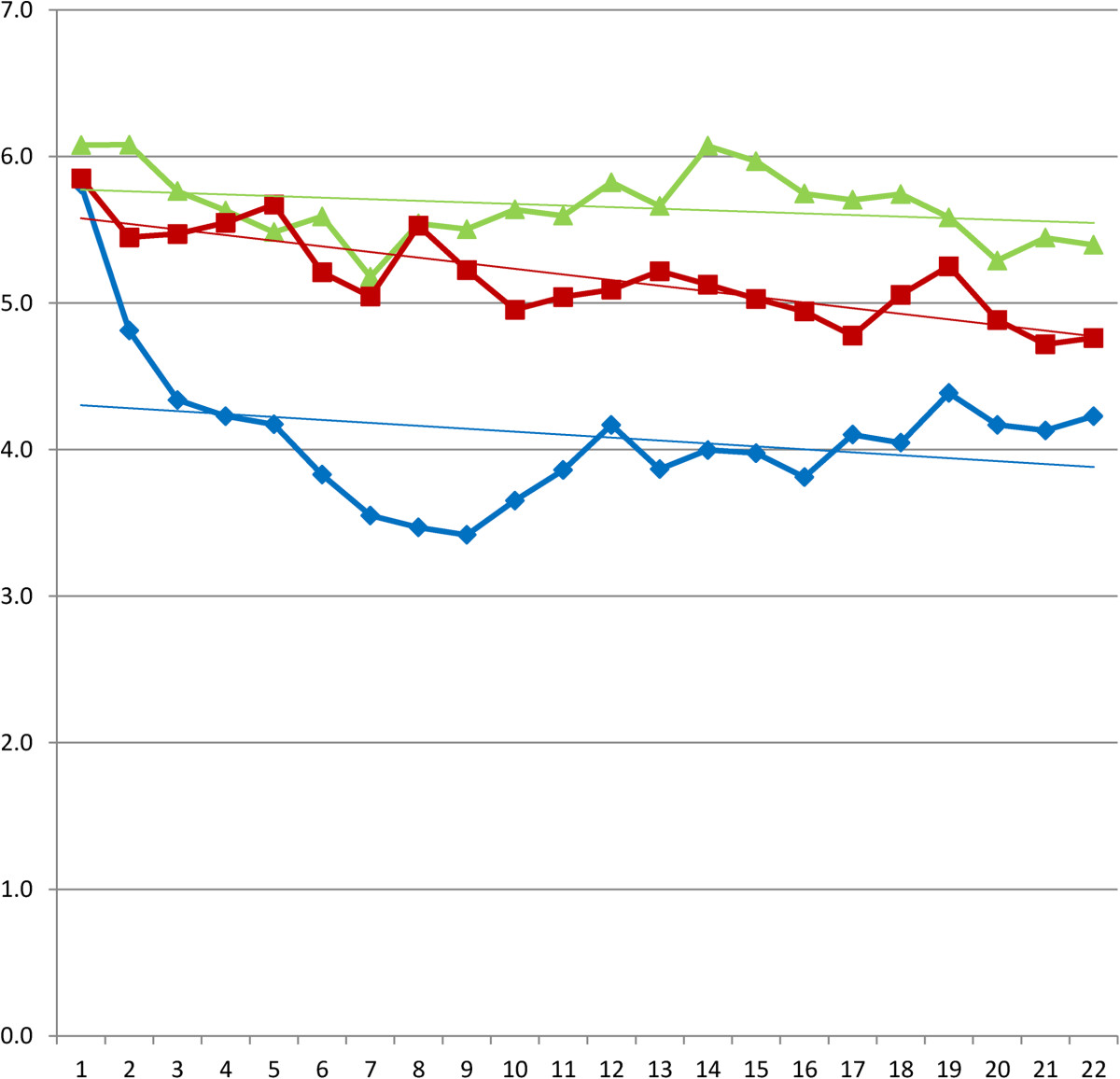
**Daily VAS scores after first injection and according linear trend lines.** Legend: (green triangle) hyaluronic acid. (red square) NaCl. (blue diamond) triamcinolone acetonide.

**Figure 3:**
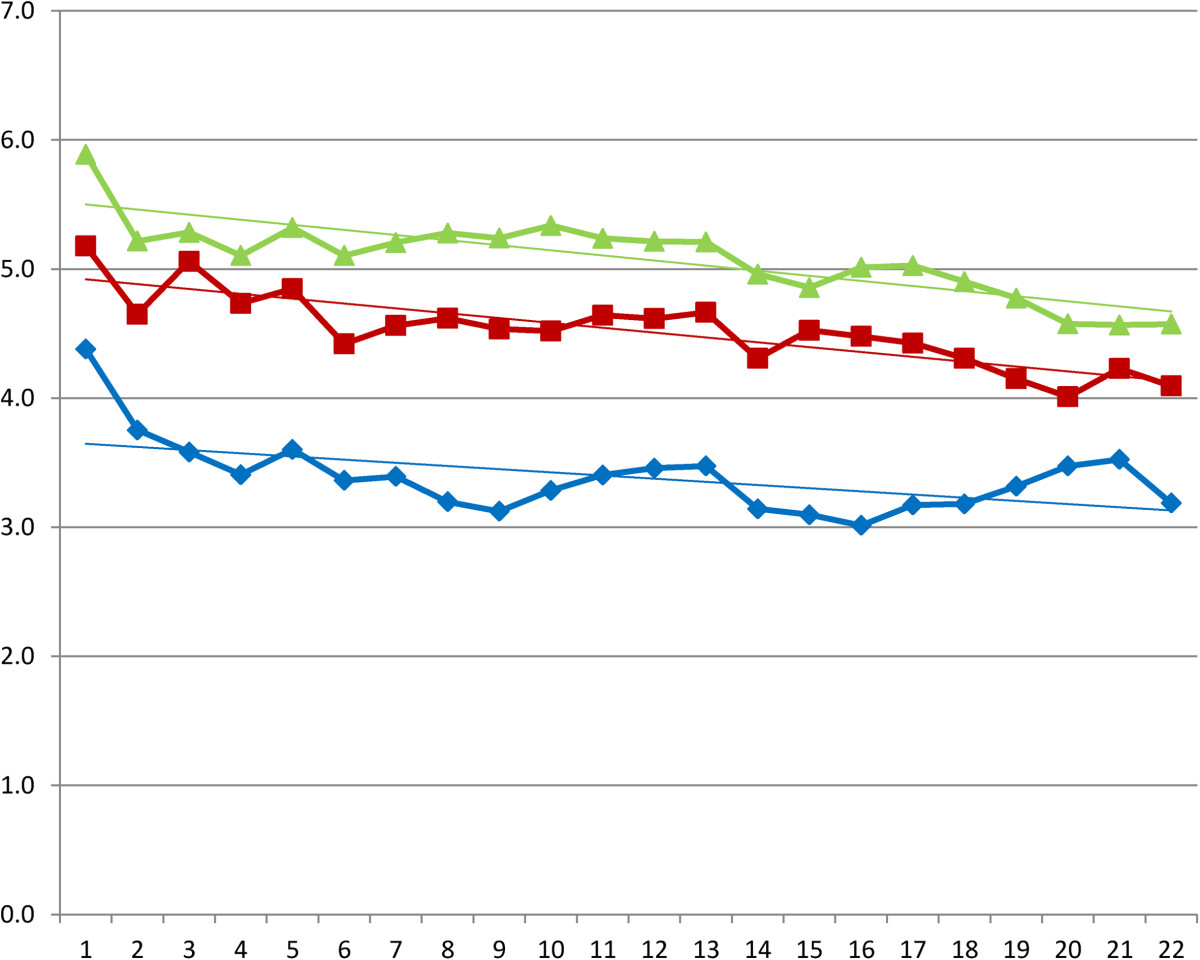
**Daily VAS scores after second injection and according linear trend lines.** Legend: (green triangle) hyaluronic acid. (red square) NaCl. (blue diamond) triamcinolone acetonide.

**Figure 4:**
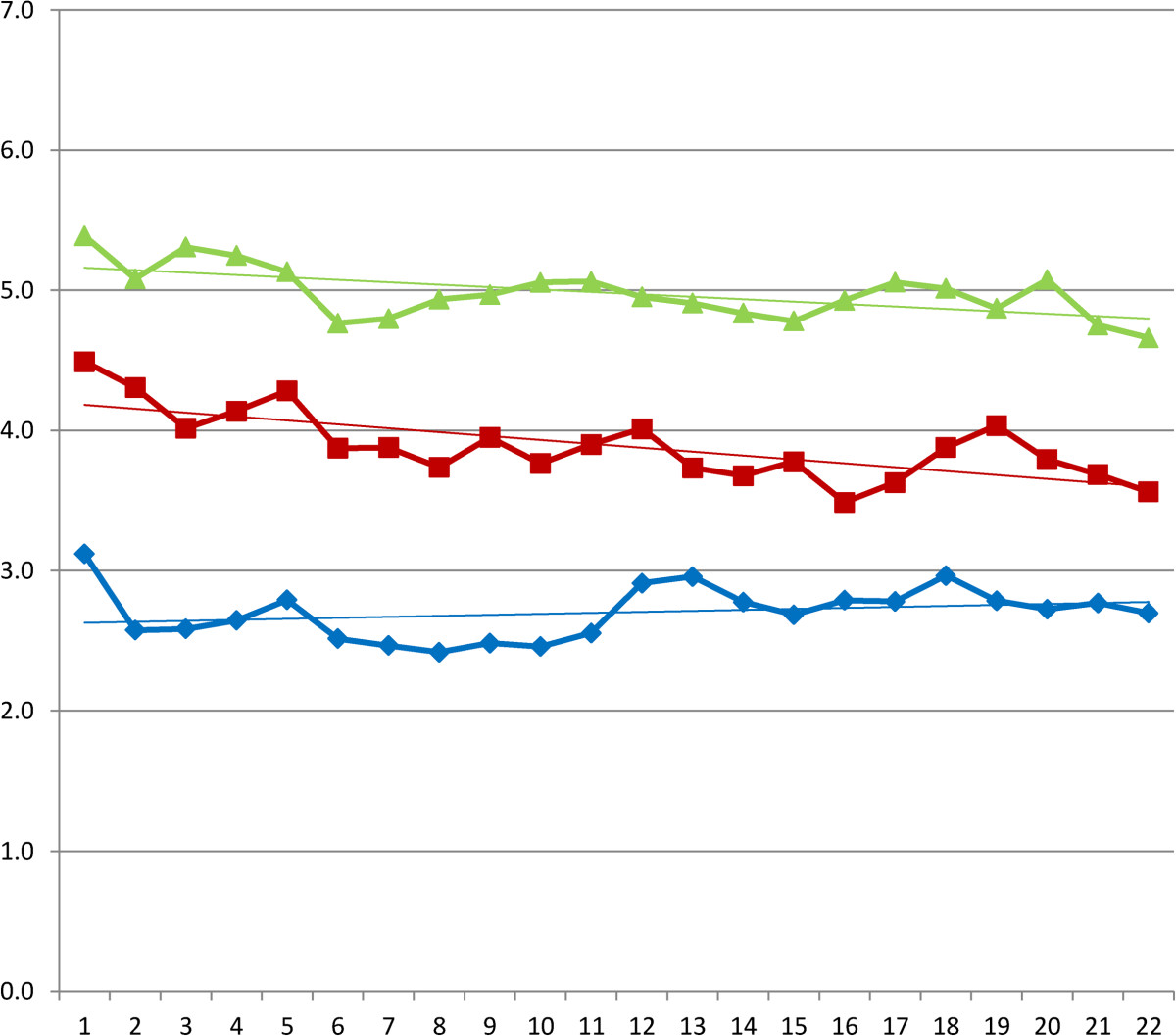
**Daily VAS scores after third injection and according linear trend lines.** Legend: (green triangle) hyaluronic acid. (red square) NaCl. (blue diamond) triamcinolone acetonide.

3% (n = 14) of the diaries was not returned, due to withdrawal from the study or lost diaries. In the hyaluronic acid group four diaries from the, first period, and two diaries of the second and third period were not returned. In the triamcinolone acetonide group two diaries in the first period, and one diary in the second and third period were not returned. For the NaCl group in both the first and second period one diary was not returned. In the third period al diaries were returned. (Table 1) Mean VAS scores and according trend lines are depicted in Figures 2, 3 and 4 and in Tables 2, 3 and 4. After the first injection a remarkable drop in VAS score is shown for the first week in the triamcinolone acetonide group, after ten days there is a slight increase in pain although the VAS score stays below the level of pain before injection at three weeks. The hyaluronic acid and NaCl group show only slight improvement in pain reduction with a less steep curve. After the second injection a slight improvement in pain is seen for the hyaluronic acid group and the NaCl group although the effect is limited compared to the effect after the first injection. After the third injection little change is shown in the VAS scores for all groups.

**Table 1:** **Lost pain diaries per group**

	***Hyaluronic acid (n = 51)***	***Triamcinolone acetonide (n = 53)***	***NaCl (n = 55)***
*Week 0 - 3*	*4*	*2*	*1*
*Week 3 - 6*	*2*	*1*	*1*
*Week 6 - 9*	*2*	*1*	*0*

**Table 2:** **Mean visual analogue scale (0–10) after the first injection**

Day	Hyaluronic acid	Triamcinolone acetonide	NaCl	Sig.
0	6,1	5,8	5,9	,845
1	6,1	4,8	5,4	,052
2	5,8	4,3	5,5	,021
3	5,6	4,2	5,5	,014
4	5,5	4,2	5,7	,008
5	5,6	3,8	5,2	,002
6	5,2	3,6	5,0	,002
7	5,5	3,5	5,5	,000
8	5,5	3,4	5,2	,000
9	5,6	3,7	5,0	,001
10	5,6	3,9	5,0	,005
11	5,8	4,2	5,1	,009
12	5,7	3,9	5,2	,002
13	6,1	4,0	5,1	,000
14	6,0	4,0	5,0	,000
15	5,7	3,8	4,9	,001
16	5,7	4,1	4,8	,008
17	5,7	4,0	5,1	,004
18	5,6	4,4	5,3	,046
19	5,3	4,2	4,9	,059
20	5,4	4,1	4,7	,018
21	5,4	4,2	4,8	,013

**Table 3:** **Mean visual analogue scale (0–10) after the second injection**

Day	Hyaluronic acid	Triamcinolone acetonide	NaCl	Sig.
0	5,9	4,4	5,2	,017
1	5,2	3,8	4,7	,022
2	5,3	3,6	5,1	,004
3	5,1	3,4	4,7	,005
4	5,3	3,6	4,8	,005
5	5,1	3,4	4,4	,004
6	5,2	3,4	4,6	,003
7	5,3	3,2	4,6	,000
8	5,2	3,1	4,5	,000
9	5,3	3,3	4,5	,001
10	5,2	3,4	4,6	,003
11	5,2	3,5	4,6	,006
12	5,2	3,5	4,7	,008
13	5,0	3,1	4,3	,003
14	4,9	3,1	4,5	,003
15	5,0	3,0	4,5	,001
16	5,0	3,2	4,4	,003
17	4,9	3,2	4,3	,005
18	4,8	3,3	4,2	,023
19	4,6	3,5	4,0	,088
20	4,6	3,5	4,2	,093
21	4,6	3,2	4,1	,006

**Table 4:** **Mean visual analogue scale (0–10) after the third injection**

Day	Hyaluronic acid	Triamcinolone acetonide	NaCl	Sig.
0	5,4	3,1	4,5	,000
1	5,1	2,6	4,3	,000
2	5,3	2,6	4,0	,000
3	5,2	2,6	4,1	,000
4	5,1	2,8	4,3	,000
5	4,8	2,5	3,9	,000
6	4,8	2,5	3,9	,000
7	4,9	2,4	3,7	,000
8	5,0	2,5	4,0	,000
9	5,1	2,5	3,8	,000
10	5,1	2,6	3,9	,000
11	5,0	2,9	4,0	,001
12	4,9	3,0	3,7	,001
13	4,8	2,8	3,7	,001
14	4,8	2,7	3,8	,001
15	4,9	2,8	3,5	,000
16	5,1	2,8	3,6	,000
17	5,0	3,0	3,9	,001
18	4,9	2,8	4,0	,001
19	5,1	2,7	3,8	,000
20	4,8	2,8	3,7	,000
21	4,7	2,7	3,6	,000

Concerning the secondary outcome measures we report significant differences in the number of persons in need of escape medication. The hyaluronic acid group and the NaCl group needed significantly more escape medication because of pain throughout the study Tables 5, 6 and 7.

**Table 5:** **Number of persons (n) in need of escape medication after the first injection**

Day	Hyaluronic acid	Triamcinolone acetonide	NaCl	Sign.
1	20	10	20	,051
2	21	11	19	,051
3	17	13	15	,514
4	15	13	17	,665
5	22	14	18	,142
6	19	14	19	,379
7	18	10	20	,061
8	21	10	20	,022
9	23	7	20	,001
10	20	8	21	,005
11	21	9	25	,002
12	20	7	19	,003
13	23	8	20	,001
14	18	7	20	,006
15	24	6	19	,000
16	21	11	15	,029
17	21	9	17	,007
18	18	10	17	,063
19	15	6	13	,011
20	15	8	14	,049
21	8	6	8	,510

**Table 6:** **Number of persons (n) in need of escape medication after the second injection**

Day	Hyaluronic acid	Triamcinolone acetonide	NaCl	Sign.
1	13	18	14	,292
2	19	6	17	,003
3	14	5	14	,031
4	18	7	17	,014
5	20	6	17	,002
6	21	6	16	,001
7	20	7	17	,005
8	22	8	13	,001
9	21	8	14	,005
10	23	6	16	,000
11	19	9	14	,037
12	18	8	17	,036
13	19	8	15	,022
14	22	8	15	,003
15	21	11	17	,047
16	18	8	16	,038
17	18	6	18	,003
18	17	6	15	,010
19	16	6	14	,011
20	12	5	14	,061
21	12	5	13	,068

**Table 7:** **Number of persons (n) in need of escape medication after the third injection**

Day	Hyaluronic acid	Triamcinolone acetonide	NaCl	Sign.
1	20	5	12	,001
2	22	6	9	,000
3	22	5	11	,000
4	18	5	10	,003
5	19	3	10	,000
6	20	3	10	,000
7	20	4	11	,000
8	16	5	11	,014
9	20	4	11	,000
10	19	4	11	,001
11	20	5	12	,001
12	19	5	11	,002
13	21	4	10	,000
14	20	7	11	,004
15	23	4	10	,000
16	24	3	10	,000
17	22	4	12	,000
18	19	3	12	,000
19	21	4	10	,000
20	18	5	10	,002
21	21	3	9	,000

Adverse effects described in the free text of the pain diaries consisted of: flushes, headache, nausea, redness at injection site and tingling sensations. 24 patients reported adverse effect, The most reported adverse effect was occurrence of headache. This occurred in 8 patients in the hyaluronic acid group, in 5 patients in the triamcinolone acetonide group and in 4 patients in the NaCl group Table 8.

**Table 8:** **Adverse events**

	Hyaluronic acid group n = 51	Triamcinolon acetonide group n = 53	NaCl group n = 55
Flushes			1
Redness at injection site	1		
Headache	8	5	4
Tingling			1
Nausea	2	2	

## Discussion

We could show a significant reduction in pain after a triamcinolone acetonide injection as we described in the results of the RCT [22]. In the data of the pain diaries we found a faster reduction and steeper downward curve in the triamcinolone acetonide group compared to the hyaluronic acid group and the NaCl group. The second injection showed a booster effect in the stepwise reduction of pain in the triamcinolone acetonide group, this effect was not seen in the hyaluronic acid and NaCl group. Little is known about the pattern of perceived pain after corticosteroid injection, by means of the recorded VAS scores we were able to give a graphic better description of this pattern. In the study of Lewis et al. the pain after corticosteroid injection in Tennis Elbow complaints was described as an increase in pain for the first day after injection and a reduction after three to four days. In our study we have not found an increase in pain as reported on the VAS score after the subacromial injections [7].

In literature most studies describe a repetition of corticosteroid injections in the treatment of shoulder disorders [19, 21].

A number of studies focus on the negative side effects of corticosteroid injections. In these studies merely the effect on the cuff tissue is described with increased damage of this tissue as summarized in the systematic review of Dean [18]. The group of Bathia however could not show a significant higher number of cuff ruptures after higher numbers of repeated corticosteroid injections [20]. The group of Mykolyzk showed a reversible effect of these tissues after 3 weeks [17]. Based on these findings there seems to be some evidence to belief that cuff tissue has the ability to recover after corticosteroid injection and that repetition of corticosteroid injections does not necessary lead to increased numbers of cuff ruptures.

There are some limitations concerning our study, the selection of patients before entering the study was performed without use of MRI or Ultrasound, this might give some bias in the selection. In general practice however most patients will be treated based on a clinical diagnosis. The results of this study therefore can be used for extrapolation to general practice.

The injections where placed without use of ultrasound guidance. A recent study however showed that there is little evidence for the need of ultrasound guided needle placement [29].

As far as we know there are no studies which show the short term accumulative effect of corticosteroids in pain reduction. One of the strengths of our study is that we were able to show a booster effect of a repeated triamcinolone acetonide injection after the second injection.

In the previously published results of the RCT we could show a significant reduction in pain on a VAS score after triamcinolone acetonide injection at three, six and twelve weeks. In the current study we were able to show the results at nine weeks after the start of the trial. Given the limited effect of reduction in pain after the third injection it is questionable whether it is necessary to administer more than two injections in case of subacromial impingement [22].

An increase in pain after corticosteroid injection is a common adverse effect [19]. Although compared to other studies in which the effect of corticosteroids was studied in the treatment of lateral epicondylitis, in our study the amount of perceived pain after triamcinolone acetonide injection was limited [7]. Patients in the hyaluronic acid group needed significantly more co-medication compared to patients in the triamcinolone acetonide group.

## Conclusions

In the first week after injection of triamcinolone acetonide we could show a fast reduction in pain. After the second injection we could show a limited booster effect of repeated triamcinolone acetonide injection. We were able to graphically show this booster effect of fast reduction in pain after the triamcinolone acetonide injection by displaying the patient perceived pain on a VAS score in the days after injection. Given the limited effect in pain reduction after the third triamcinolone acetonide injection there might be evidence to reduce the total number of repeated triamcinolone acetonide injections to two injections.

Compared to Hyaluronic acid and NaCl injections, triamcinolone acetonide injections showed a fast and effective reduction in pain on a VAS score already after a limited number of injections.

## Authors’ original submitted files for images

Below are the links to the authors’ original submitted files for images. Authors’ original file for figure 1 Authors’ original file for figure 2 Authors’ original file for figure 3 Authors’ original file for figure 4

## References

[1] Greving K, Dorresteijn O, Winters JC, Groenhof F, van der Meer K, Stevens M, Diercks RL (2012). Incidence, prevalence, and consultation rates of shoulder complaints in general practice. Scand J Rheumatol.

[2] Picavet HSJ, Schouten JSAG (2003). Musculoskeletal pain in the Netherlands: prevalences, consequences and risk groups, the DMC3-study. Pain.

[3] Michener LA, McClure PW, Karduna AR (2003). Anatomical and biomechanical mechanisms of subacromial impingement syndrome. Clin Biomech.

[4] Blair B, Rokito AS, Cuomo F, Jarolem K, Zuckerman J (1996). Efficacy of injections of corticosteroids for subacromial impingement syndrome. J Bone Joint Surg Am.

[5] Winters JC, van der Windt DAWM, Spinnewijn WEM, De Jogh AC, Van der Heijden GJMG, Buis PAJ, Boeke AJP, Feleus A, Geraedts JJXR (2008). NHG-Standaard Schouderklachten. Huisarts en Wetenschap.

[6] Coombes BK, Bisset L, Vicenzino B (2010). Efficacy and safety of corticosteroid injections and other injections for management of tendinopathy: a systematic review of randomised controlled trials. Lancet.

[7] Lewis M, Hay E, Paterson SM, Croft P (2005). Local steroid injections for tennis elbow: does the pain get worse before it gets better?: Results from a randomized controlled trial. Clin J Pain.

[8] Crawshaw DP, Helliwell PS, Hensor EM, Hay EM, Aldous SJ, Conaghan PG (2010). Exercise therapy after corticosteroid injection for moderate to severe shoulder pain: large pragmatic randomised trial. BMJ.

[9] Shibata Y, Midorikawa K, Emoto G, Naito M (2001). Clinical evaluation of sodium hyaluronate for the treatment of patients with rotator cuff tear. J Shoulder Elbow Surg.

[10] Itokazu M, Matsunaga T (1995). Clinical evaluation of high-molecular-weight sodium hyaluronate for the treatment of patients with periarthritis of the shoulder. Clin Ther.

[11] Funk L (2005). P153 Hyaluronan vs. steroid injection for subacromial impingement of the shoulder. Osteoarthr Cartil.

[12] Chou WY, Ko JY, Wang FS, Huang CC, Wong T, Wang CJ, Chang HE (2010). Effect of sodium hyaluronate treatment on rotator cuff lesions without complete tears: a randomized, double-blind, placebo-controlled study. J Shoulder Elbow Surg.

[13] Iwata H (1993). Pharmacologic and clinical aspects of intraarticular injection of hyaluronate. Clin Orthop Relat Res.

[14] Kuiper-Geertsma DG, Bijlsma JW (2000). [Intra-articular injection of hyaluronic acid as an alternative option to corticosteroid injections for arthrosis]. Ned Tijdschr Geneeskd.

[15] Eustace JA, Brophy DP, Gibney RP, Bresnihan B, FitzGerald O (1997). Comparison of the accuracy of steroid placement with clinical outcome in patients with shoulder symptoms. Ann Rheum Dis.

[16] Tillander B, Franzen LE, Karlsson MH, Norlin R (1999). Effect of steroid injections on the rotator cuff: an experimental study in rats. J Shoulder Elbow Surg.

[17] Mikolyzk DK, Wei AS, Tonino P, Marra G, Williams DA, Himes RD, Wezeman FH, Callaci JJ (2009). Effect of corticosteroids on the biomechanical strength of rat rotator cuff tendon. J Bone Joint Surg.

[18] Dean BJF, Lostis E, Oakley T, Rombach I, Morrey ME, Carr AJ (2014). The Risks and Benefits of Glucocorticoid Treatment for Tendinopathy: A Systematic Review of the Effects of Local Glucocorticoid on Tendon. Semin Arthritis Rheum.

[19] Buchbinder R, Green S, Youd JM (2003). Corticosteroid injections for shoulder pain. Cochrane Database Syst Rev.

[20] Bhatia M, Singh B, Nicolaou N, Ravikumar KJ (2009). Correlation between rotator cuff tears and repeated subacromial steroid injections: a case-controlled study. Ann R Coll Surg Engl.

[21] Gaujoux-Viala C, Dougados M, Gossec L (2009). Efficacy and safety of steroid injections for shoulder and elbow tendonitis: a meta-analysis of randomised controlled trials. Ann Rheum Dis.

[22] Penning LIF, de Bie RA, Walenkamp GHIM (2012). The effectiveness of injections of hyaluronic acid or corticosteroid in patients with subacromial impingement: a three-arm randomised controlled trial. J Bone Joint Surg.

[23] MacGregor AJ (1995). Classification criteria for rheumatoid arthritis. Baillieres Clin Rheumatol.

[24] van der Linden S, Valkenburg HA, Cats A (1984). Evaluation of diagnostic criteria for ankylosing spondylitis. A proposal for modification of the New York criteria. Arthritis Rheum.

[25] Rowe CR (1988). Injection technique for the shoulder and elbow. Orthop Clin North Am.

[26] Huskisson EC, Melzack R (1983). Visual Analogue Scales. Pain Measurement and Assessment.

[27] de Wit R, van Dam F, Hanneman M, Zandbelt L, van Buuren A, van der Heijden K, Leenhouts G, Loonstra S, Huijer Abu-Saad H (1999). Evaluation of the use of a pain diary in chronic cancer pain patients at home. Pain.

[28] Neer CS (1983). Impingement lesions. Clin Orthop Relat Res.

[29] Ekeberg OM, Bautz-Holter E, Tveita EK, Juel NG, Kvalheim S, Brox JI (2009). Subacromial ultrasound guided or systemic steroid injection for rotator cuff disease: randomised double blind study. BMJ.

[CR30] The pre-publication history for this paper can be accessed here:http://www.biomedcentral.com/1471-2474/15/352/prepub

