# Supplementary figures and images for: Subacromial triamcinolone acetonide, hyaluronic acid and saline injections for shoulder pain an RCT investigating the effectiveness in the first days
Source: BMC Musculoskelet Disord. 2014 Oct 23;15:352. doi: 10.1186/1471-2474-15-352 (PMC4213555; doi:10.1186/1471-2474-15-352)

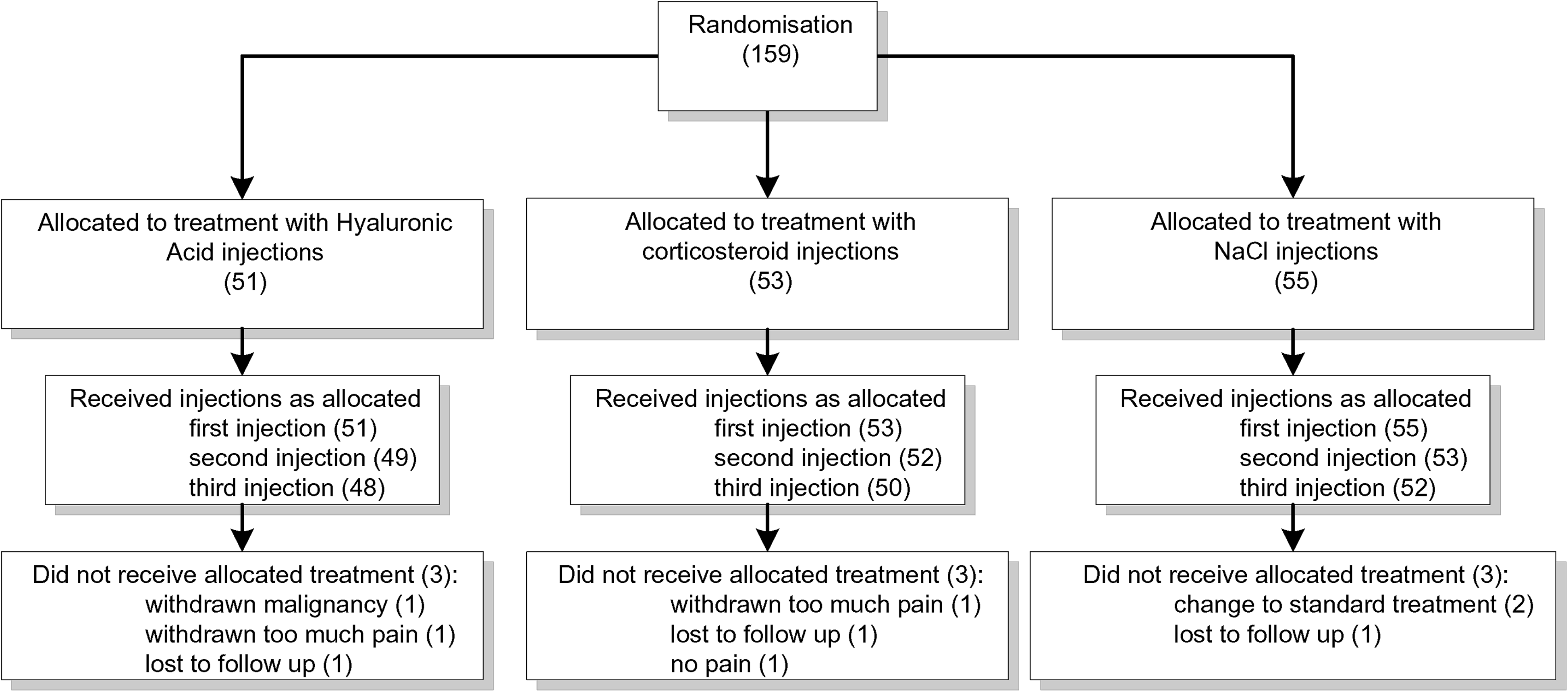

Supplement: Supplementary file 1 — Authors’ original file for figure 1 [file 12891_2014_2286_MOESM1_ESM.tif]

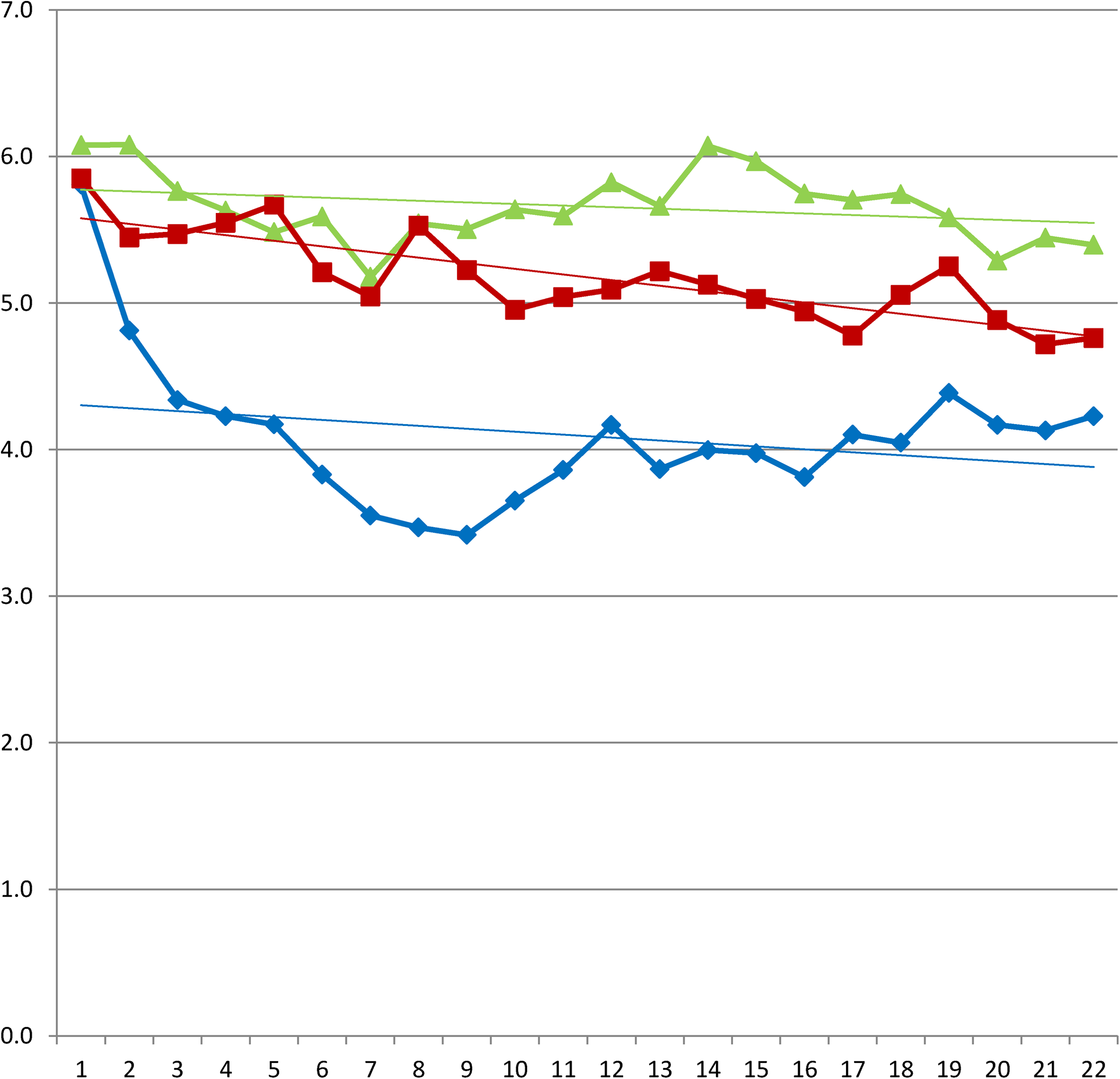

Supplement: Supplementary file 2 — Authors’ original file for figure 2 [file 12891_2014_2286_MOESM2_ESM.tif]

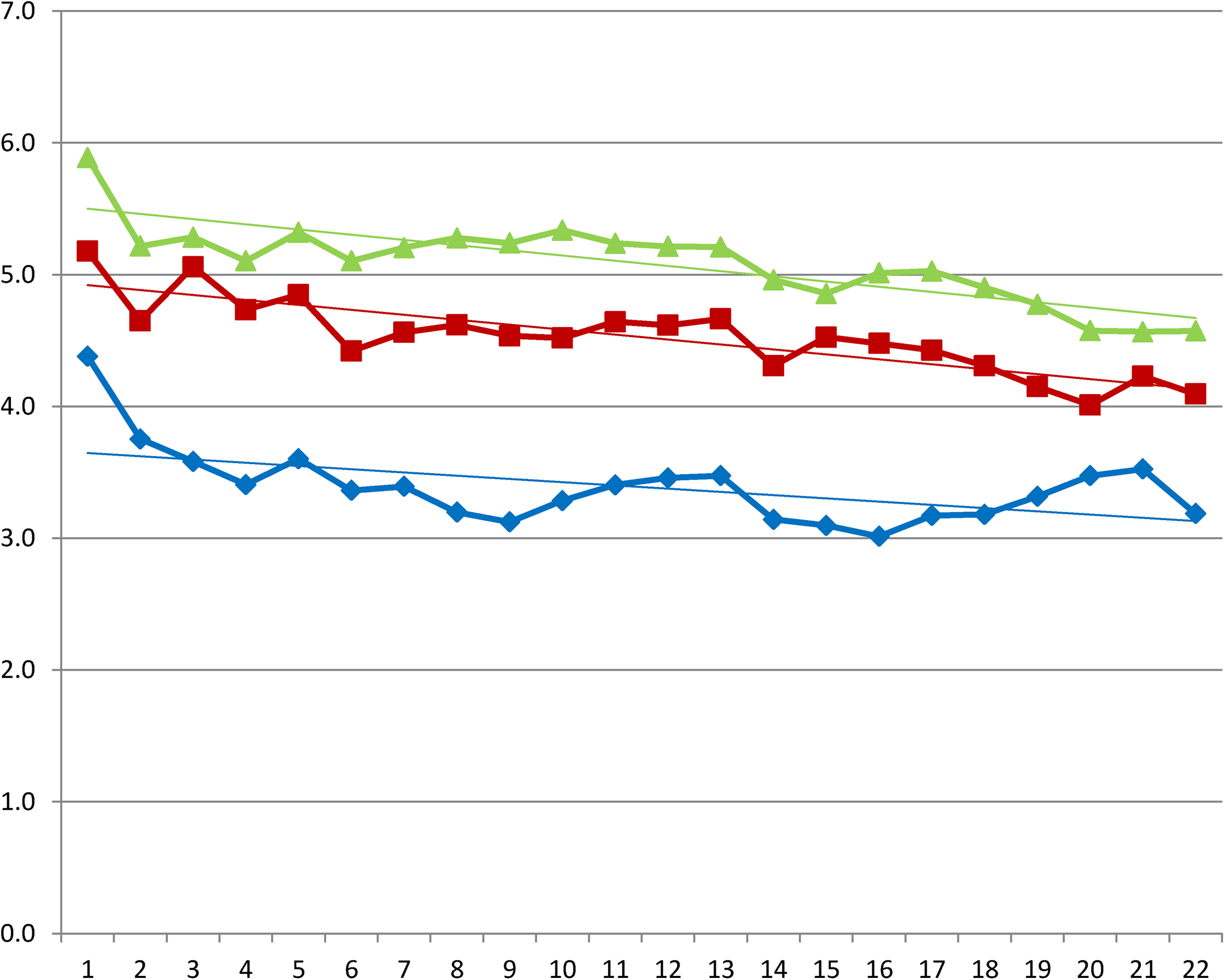

Supplement: Supplementary file 3 — Authors’ original file for figure 3 [file 12891_2014_2286_MOESM3_ESM.tif]

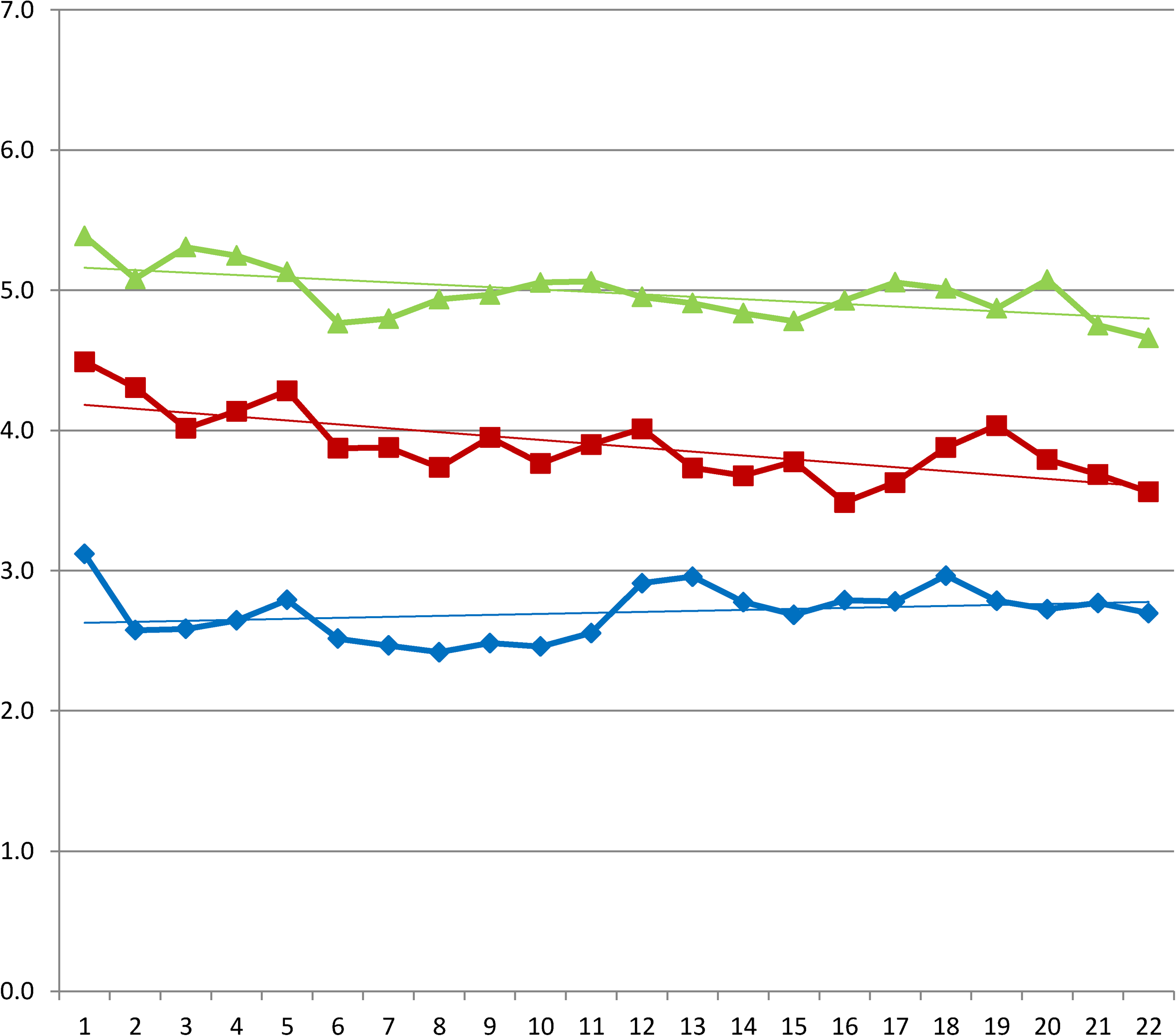

Supplement: Supplementary file 4 — Authors’ original file for figure 4 [file 12891_2014_2286_MOESM4_ESM.tif]
